# Treatment of efferent loop syndrome after pancreatoduodenectomy with a fully covered self‐expandable metal stent: A case report

**DOI:** 10.1002/deo2.156

**Published:** 2022-07-29

**Authors:** Jun Wu, Cui Chen, Bing Hu

**Affiliations:** ^1^ Department of Gastroenterology, Eastern Hepatobiliary Hospital Naval Military Medical University Shanghai China

**Keywords:** case report, efferent loop syndrome, endoscopic treatment, metal stent, pancreatoduodenectomy

## Abstract

Efferent loop syndrome is a very rare complication following pancreatoduodenectomy. The treatment of efferent loop syndrome varies depending on the cause of the syndrome. Conservative treatment methods, including nasogastric drainage and enteral nutrition, are adopted that are effective in most of patients; however, surgical treatment is usually required in patients with complete loop obstruction. Herein, we report a case of severe efferent loop obstruction that occurred after pancreatoduodenectomy, which was refractory to conservative treatment and successfully resolved by insertion of a fully covered self‐expandable metal stent.

## INTRODUCTION

Pancreatoduodenectomy (PD) has been the standard modality for surgical treatment of periampullary and pancreatic carcinoma.^1^ Efferent loop syndrome (ELS) is a very rare complication following PD, which usually occurs within the first two weeks following surgery; the causes of ELS include anastomotic edema, bowel adhesion, greater omentum edema, inflammatory mass compression, or poor reconstruction during surgery.^2^ Herein, we report a case of severe efferent loop obstruction occurred after PD, and the possible cause of it maybe bowel adhesion and abnormal blood supply. The efferent loop obstruction was successfully resolved by the insertion of a fully covered self‐expandable metal stent (FCSEMS).

## CASE REPORT

**FIGURE 1:**
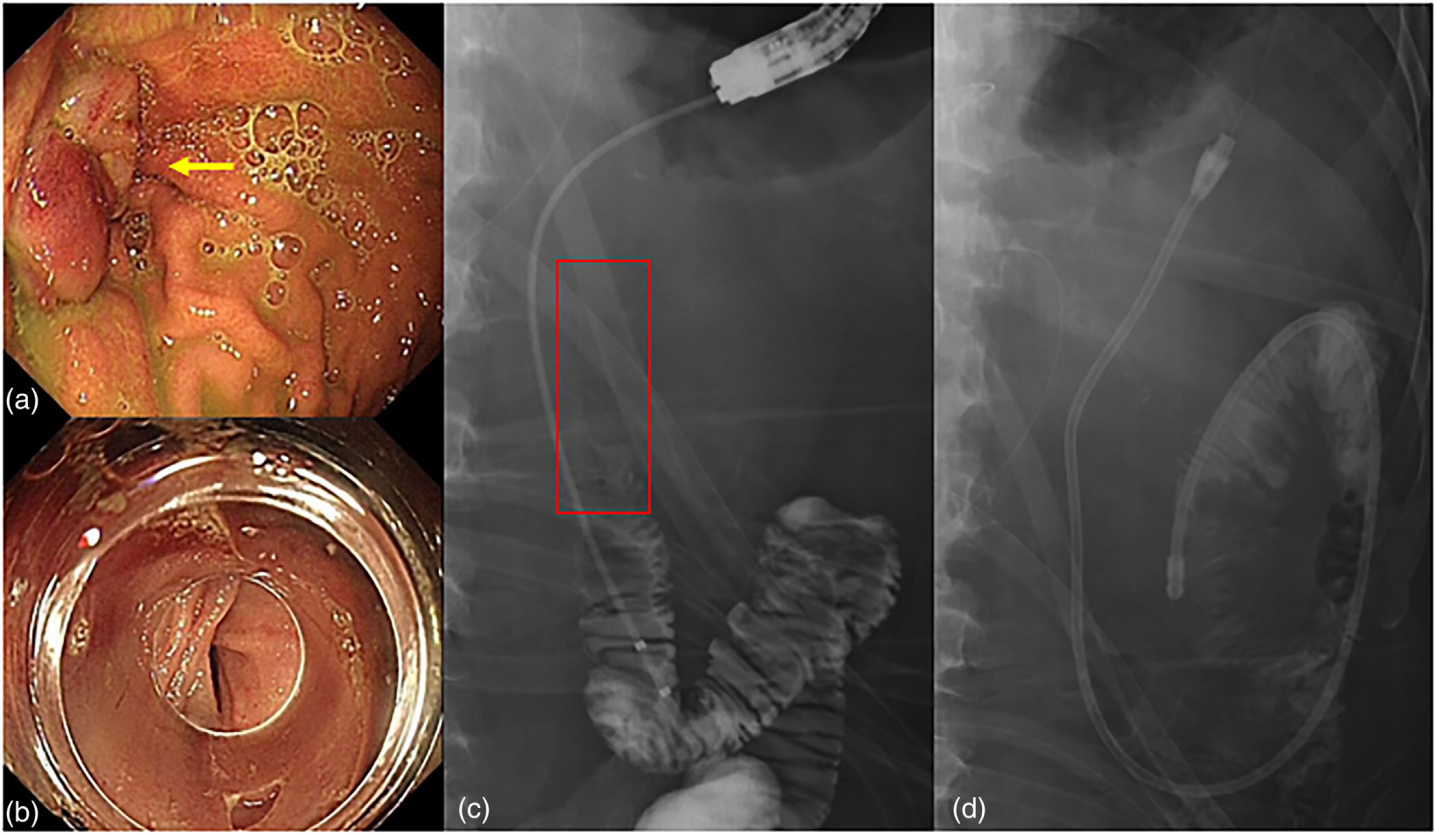
The first endoscopic intervention process. (a) Gastroscopy showed anastomotic stoma is severely edematous. (b) The intestine of efferent loop was narrow and edematous. (c) Enterography showed stenosis of efferent loop approximately 3 cm below the site of anastomosis. (d) A three‐lumen gastrojejunal tube was placed across the anastomosis and intestinal stricture

A 70‐year‐old male patient, diagnosed with duodenal papillary carcinoma, underwent pancreaticoduodenectomy in our hospital. The patient recovered well and started orally; however, 10 days later, he developed abdominal discomfort and experienced episodes of bilious vomiting. A large amount of yellowish bile was extracted after gastrointestinal decompression. Plan abdominal radiography and laboratory tests did not show remarkable findings. Gastroscopy showed that anastomotic stoma is severely edematous (Figure 1a), the afferent loop could be entered easily, the lumen of the afferent loop was normal, but the efferent loop could not be entered. With the aid of transparent cap, the endoscope could barely enter the efferent loop. The intestine of efferent loop was narrow and edematous (Figure 1b), and the endoscope could not pass through the stenosis. The narrowed loop did not appear to have any abnormal mucosal lesions. Enterography showed stenosis of efferent loop approximately 3 cm below the site of anastomosis (Figure 1c); according to the surgeon's requirement, a three‐lumen gastrojejunal tube which supplies gastrointestinal decompression and enteral nutrition was placed across the anastomosis and intestinal stricture (Figure 1d).

**FIGURE 2:**
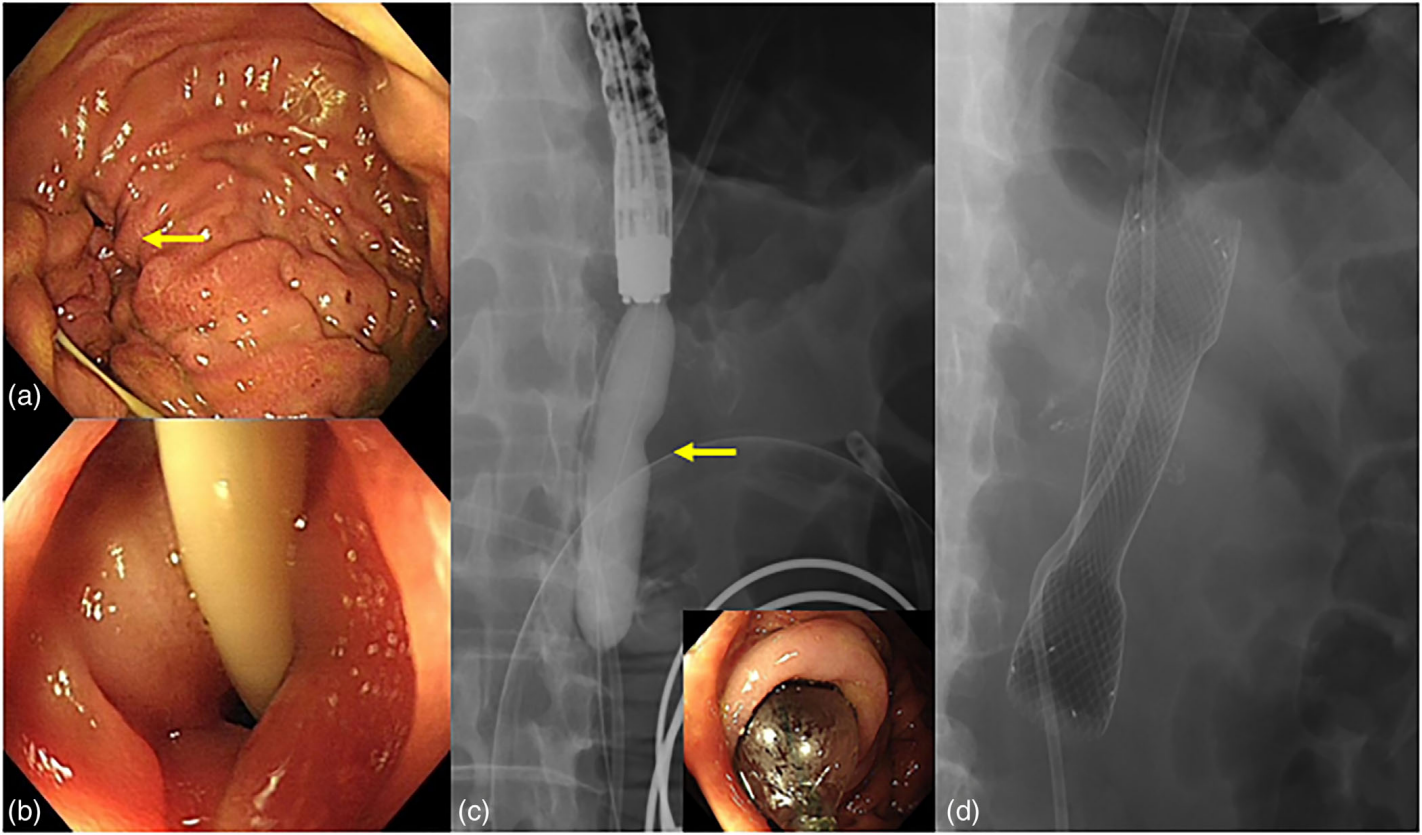
The second endoscopic intervention process. (a) Gastroscopy showed anastomotic edema was significantly relieved. (b) The intestine of efferent loop was still narrow. (c) Endoscopic pneumatic balloon dilatation is shown, and thick yellow arrow indicated the stenosis area. (d) A fully covered metal stent was inserted through the stenosis of efferent loop and over the guide wire under fluoroscopy

**FIGURE 3:**
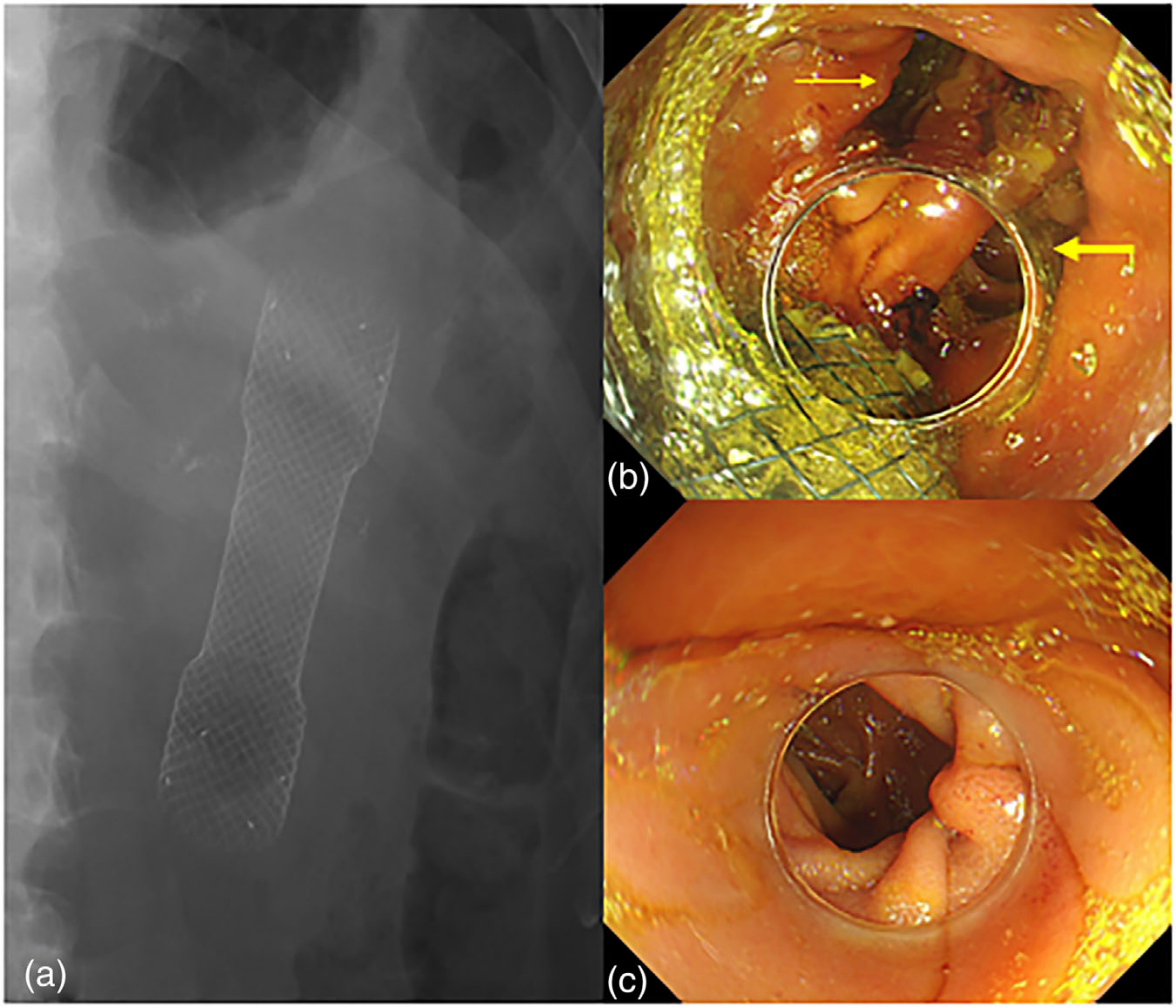
The third endoscopic intervention process. (a) Abdominal fluoroscopy showed that the stent was in good position and the stricture was relieved at 1 month after stent insertion. (b) The stent was completely displaced into the gastric cavity, thick yellow arrow indicated the orifice of afferent loop, and thin yellow arrow indicated the orifice of efferent loop. (c) The stenosis of efferent loop was significantly relieved

One month later, the patient came back to our hospital; he complained that a large amount of bile (600–800 ml) was attracted every day, and he still could not eat anything. Gastroscopy was performed, anastomotic edema was significantly relieved (Figure 2a), but the intestine of efferent loop was still narrow (Figure 2b), and endoscope could not pass through the stenosis. Endoscopic pneumatic balloon dilatation (CRE Balloon, Boston Scientific; 13.5 mm; 40 psi for 1 min) over the guide wire and under endoscopic view was immediately performed (Figure 2c); however, there was no significant improvement in stenosis. Subsequently, a fully covered metal stent (Evolution Esophageal Stent, 20 mm × 8 cm; Cook) was inserted through the efferent loop stenosis and over the guide wire under fluoroscopy (Figure 2d). After stent placement, the drainage of gastric tube decreased significantly, and patient had no abdominal distension and vomiting after eating. He was discharged 5 days later. At 1 month, the patient's abdominal fluoroscopy showed that the stent was in good position and the stricture was relieved (Figure 3a). Two months after stent placement, we informed the patient to remove the stent. Gastroscopy showed that the stent was completely displaced into the gastric cavity (Figure 3b); it was removed with grasping forceps successfully. The stenosis of efferent loop was significantly relieved (Figure 3c), and the endoscope could get to distal small intestine smoothly.

The patient has been followed up for more than 1 year; he had a good diet and no other symptoms.

## DISCUSSION

Up to 1% of patients who undergo partial gastrectomy will experience afferent loop syndrome, and compared with afferent loop syndrome, very little data have been reported regarding ELS making it much rarer.^2^ The mechanical problem that occurs in ELS and afferent loop syndrome is the obstruction of either efferent or afferent limb, respectively. These two syndromes may be difficult to differentiate due to similar symptoms. The prognosis is good for patients who receive a prompt diagnosis and undergo timely treatment. The treatment of ELS varies depending on the cause of the syndrome; in majority of patients, conservative treatment methods, including nasogastric drainage and enteral nutrition, are suggested that are effective^3^; however, surgical treatment is usually required in patients with complete loop obstruction.^4^ This case received conservative treatment first, but the effect was not ideal. For the persistent intestinal stenosis, there was indication for further intervention under endoscopy or surgery. In recent years, endoscopic techniques, including metal stent insertion, endoscopic stricturotomy, endoscopic ultrasound guided gastrojejunostomy, and so on, have been widely used in the treatment of various benign and malignant gastrointestinal strictures.^5^ However, in the literature, information regarding the treatment of ELS is limited.^6^, ^7^

According our previous experience, small size balloon (12–15 mm) is used to dilate the stenosis, which is convenient to accurately judge the location and degree of stenosis. For short time after surgery, in order to avoid anastomotic leakage and intestinal perforation caused by dilation, the large size balloon (15–18 mm) was not used. We originally planned to only use balloon to dilate the stenosis. However, the stenosis was serious. When the balloon was inflated to 13.5 mm and maintained for 60 s, the stenosis still could not be relieved. Therefore, we did not expand to a larger diameter to avoid serious complications. Combined use of a metal stent could take advantage of the gradual expansion of stent to achieve sustained and slow expansion. The result confirmed that treatment with FCSEMS was safe and effective, without any related complications, and the stenosis was completely relieved.

There is a correlation between stent retention time and curative effect; if the time is too short, the stenosis is relatively easy to recur. Previous studies reported that the stent retention time ranged from 7 to 60 days.^8^ The stent in this case was placed for 2 months according to treatment arrangement, and after removing the stent, the stenosis was completely relieved.

Early stent migration will also affect the curative effect, and as we know, FCSEMS is prone to displace.^8^ Therefore, we closely monitored the position of stent; at 1 month after stent insertion, the stent was fully expanded and well positioned. So, there was no early recurrence of stenosis after stent removement during follow‐up. However, the stent was displaced completely at 2 months. Stent migration might cause significant complications. If stent migrates to the anal side, intestinal obstruction or perforation could be caused. Therefore, stent migration is still the point of our clinical attention and research in future. In recent years, in terms of preventing stent migration, novel stent (lumen‐apposing fully covered metal stent) or over‐the‐scope‐clip has been used and achieved very good results.^9^

In conclusion, in patients with severe ELS requiring surgical treatment, FCSEMS could be considered as a safe and effective treatment option for relieving severe efferent loop obstruction after surgery.

## CONFLICT OF INTEREST

The authors declare that they have no conflict of interest.

## FUNDING INFORMATION

Youth Development Project of the Peak Discipline of Nursing of Naval Military Medical University, Grant Number: 18QPFH25; Shanghai Science and Technology Innovation Action Plan Medical Field Project, Grant Number: 16411951700.

## AUTHOR CONTRIBUTIONS

Jun Wu and Cui Chen performed data extraction, and Jun Wu and Bing Hu wrote the paper. Bing Wu and Cui Chen contributed equally to this work.

## INFORMED CONSENT

A written informed consent was obtained from the patient for the publication of this case report and the accompanying images.
